# A Note on Spatial Averaging and Shear Stresses Within Urban Canopies

**DOI:** 10.1007/s10546-017-0321-7

**Published:** 2017-11-23

**Authors:** Zheng-Tong Xie, Vladimir Fuka

**Affiliations:** Faculty of Engineering and the Environment, University of Southampton, Southampton, SO17 1BJ UK

**Keywords:** Comprehensive spatial average, Effective total shear stress, Intrinsic spatial average, One-dimensional column urban model, Vertical momentum flux

## Abstract

One-dimensional urban models embedded in mesoscale numerical models may place several grid points within the urban canopy. This requires an accurate parametrization for shear stresses (i.e. vertical momentum fluxes) including the dispersive stress and momentum sinks at these points. We used a case study with a packing density of 33% and checked rigorously the vertical variation of spatially-averaged total shear stress, which can be used in a one-dimensional column urban model. We found that the intrinsic spatial average, in which the volume or area of the solid parts are not included in the average process, yield greater time–spatial average of total stress within the canopy and a more evident abrupt change at the top of the buildings than the comprehensive spatial average, in which the volume or area of the solid parts are included in the average.

## Introduction

The horizontal resolution of operational mesoscale models is now around 1 km for a computational domain of a few hundreds of kilometre square including urban areas. It is urgently required to have a more accurate parametrization of the airflow within the urban canopy ‘representing aggregate effects of heterogeneous urban elements as subgrid processes’ (Fernando 2010), so as to estimate vertical profiles of effective total shear stress, turbulent shear stress, dispersive shear stress and vertical distributions of drag coefficient, heat source and scalar sources at a few levels per average building height in one-dimensional column urban models (e.g. Martilli 2002; Kondo et al. 2005; Santiago and Martilli 2007; Masson and Seity 2009; Martilli and Santiago 2010; Husain et al. 2013; Gutierrez et al. 2015; Sharma et al. 2017). It is even more important for the parametrization over an urban area with densely placed high-rise buildings. Note that the effective total shear stress is the sum of all shear-stress components including the converted form (pressure) drag and viscous drag due to the buildings, denoted by total shear stress or total stress thereafter.

The spatial-averaging procedure needs to be designed to estimate these shear stresses (i.e. vertical momentum fluxes) including dispersive stress and momentum sinks (i.e. drag) in one-dimensional column urban models. We hereafter consider that the term ‘vertical momentum flux’ ($\hbox {m}^2$ $\hbox {s}^{-2}$) is equivalent to the term ‘shear stress’, while the term ‘total vertical momentum flux of the computational domain’ (unit of $\hbox {m}^4$ $\hbox {s}^{-2}$) is equivalent to the term ‘total shear force’. The term ‘dispersive flux’ or ‘dispersive stress’ was introduced about half a century ago (e.g. Gray 1975; Wilson and Shaw 2007). Wilson and Shaw (2007) used dispersive stress to develop a one-dimensional numerical model for airflow within vegetative canopies. The dispersive stress is the vertical momentum flux extracted from the airflow at a certain height to the level below or above, which is due to spatial variations of the local time-averaged velocities calculated using spatial averaging over a horizontal plane. These are analogues of the Reynolds stresses, while the latter are cross-correlations of turbulent fluctuations usually calculated using time averaging at one point.

There is a debate on whether the averaging volume excludes or includes the solid parts. The former is represented by

1 $$\begin{aligned} \left\langle \phi \right\rangle _I (z)=\frac{1}{S_f}\int _{S_f} \phi (x,y,z) \text {d}x \text {d}y, \end{aligned}$$

where $\phi$ is a flow quantity, such as velocity, stress, heat flux and scalar flux, $\langle \,\rangle$ denotes spatial averaging, $S_f$ is the fluid area. $\left\langle \phi \right\rangle _I$ is known as the intrinsic spatial average (ISA) (e.g. Slattery 1999), which is widely used in the literature, e.g., Gray (1975), Wilson and Shaw (2007), Raupach and Shaw (1982), Raupach et al. (1986), Finnigan (2000), Coceal et al. (2006), Nikora et al. (2007) and Xie et al. (2008).

The averaging volume that includes the solid parts is given by

2 $$\begin{aligned} \left\langle \phi \right\rangle _c (z)=\frac{1}{S_c}\int _{S_f} \phi (x,y,z) \text {d}x \text {d}y, \end{aligned}$$

where $S_c$ is the total area including that occupied by solid; $\left\langle \phi \right\rangle _c$ is defined here as the comprehensive spatial average (CSA).

So far very little of the literature reports use of the CSA. Yuan and Piomelli (2014) chose the CSA to analyze the momentum flux budgets in turbulence within and above a group of randomly rotated ellipsoids. Their numerical data were obtained using direct numerical simulation (DNS) with an immersed boundary method (IBM) to represent the ellipsoids, and the spatially-averaged vertical profiles of components of the vertical momentum flux show excellent consistency below and immediately above the roughness crest.

Recently we used large-eddy simulation (LES) to calculate flow over a group of aligned cuboids with a packing density of 33% (Castro et al. 2016). When using the ISA, we noticed in this case study that, (1) the time- and spatially-averaged vertical profile of total shear stress shows an abrupt change at the canopy height; (2) the total shear stress immediately below the canopy height is much greater than that immediately above the canopy; (3) the magnitude of the total shear stress within the canopy calculated from the ISA approach is much greater than that from the CSA approach. This may cause serious problems for the parametrization of momentum flux, drag coefficient, heat sources and scalar sources for a one-dimensional column urban model in mesoscale models if no attention is paid. This Note focuses on this issue and attempts to provide a suggestion.

## Case Study

**Fig. 1:**
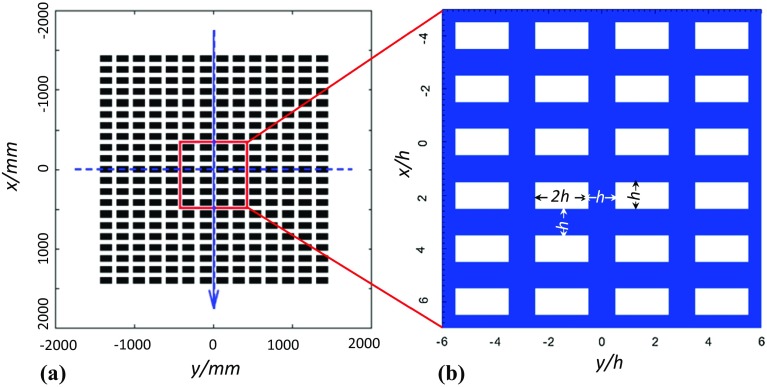
Arrays of aligned cuboids with dimensions 2*h* (length) $\times$ *h* (width) $\times$ *h* (height). All spacings between the cuboids are *h*, with $h = 70$ mm. The Reynolds number based on cuboid height and the velocity at that height in the upstream boundary layer in the wind tunnel was about 7400. **a** wind-tunnel model. **b** Numerical model in a computational domain 12*h* ($L_x$) $\times$ 12*h* ($L_y$) $\times$ 12*h* ($L_z$) with periodic boundary conditions in the horizontal directions

We focus on a case study (see Fig. 1b) that simulates flow over a group of aligned cuboids with a packing density of 33% (Castro et al. 2016). Large-eddy simulation was used with governing equations,

3 $$\begin{aligned} \frac{\partial u_i}{\partial t} + \frac{\partial u_iu_j}{\partial x_j} = -\frac{\partial }{\partial x_i}\left( \frac{p}{\rho }\right) +\frac{\partial }{\partial x_j}\left( \nu \frac{\partial u_i}{\partial x_j}\right) +\frac{\partial }{\partial x_j}\left( -\widehat{u''_iu''_j}\right) - \delta _{i1}\frac{\partial }{\partial x_i}\left( \frac{P}{\rho }\right) . \end{aligned}$$

The resolved velocity and pressure are respectively given by $u_i$ and *p* with $u (u_1)$, $v (u_2)$ and $w (u_3)$ the streamwise, lateral and vertical velocity components respectively; $u''_i$ is the subgrid-scale (SGS) velocity. The last term $- \delta _{i1}\frac{\partial }{\partial x_i}\left( \frac{P}{\rho }\right)$ is the specified driving body force, $\rho$ and $\nu$ are the density and kinematic viscosity of the fluid, $\widehat{~}$ denotes subgrid filtering, $-\widehat{u''_iu''_j}$ is the SGS stress and is handled using the mixing time scale model (Inagaki et al. 2005).

The domain length and width are significantly less than those of the wind-tunnel model (Fig. 1a) placed in a simulated boundary layer. The domain height is 12*h*, which is slightly less than the boundary-layer height 14*h* in the wind tunnel. The LES data are validated using the wind-tunnel measurements in Castro et al. (2016).

On the top of the domain, stress-free boundary conditions are specified, i.e.,

4 $$\begin{aligned} \frac{\partial u}{\partial z}=\frac{\partial v}{\partial z}=0; \quad w=0, \end{aligned}$$

while periodic boundary conditions are specified in the streamwise and lateral directions. A constant pressure gradient $- \delta _{i1}\frac{\partial }{\partial x_i}\left( \frac{P}{\rho }\right)$ is used to drive the flow, which can also be seen as a body force in the fluid. Using periodic boundary conditions in the horizontal directions and knowing the exact driving force are the key to assessing the budget of the vertical momentum flux, and this is our focus herein. The cuboid surfaces and the floor are defined as non-slip walls, and are expected to contribute to 100% of the total drag, which is equal to the integration of the driving body force.

## Spatially-Averaged Stresses

Using the ISA over the whole ($x{-}y$) plane (Fig. 1), a resolved instantaneous flow quantity $\phi$ in LES can be further decomposed into the space-time average $\left\langle \overline{\phi }\right\rangle$, spatial variation of the time average $\tilde{\phi }$, and resolved turbulence fluctuation $\phi '$ which is the deviation of the resolved instantaneous quantity $\phi$ from the time average $\overline{\phi }$,

5a $$\begin{aligned} \phi =\left\langle \overline{\phi }\right\rangle +\tilde{\phi }+\phi ', \end{aligned}$$

and

5b $$\begin{aligned} \overline{\phi }=\left\langle \overline{\phi }\right\rangle +\tilde{\phi }. \end{aligned}$$

Applying the ISA to the time-averaged *u*-momentum equation of Eq. 3 for the ($x{-}y$) planes of the computation domain in Fig. 1b, we obtain,

6 $$\begin{aligned} -\left\langle \frac{\partial P}{\rho \partial x_1}\right\rangle -\left\langle \frac{\partial \overline{p}}{\rho \partial x_1}\right\rangle +\left\langle \frac{\partial }{\partial x_j}\left( \nu \frac{\partial \overline{u_i}}{\partial x_j}\right) \right\rangle -\left\langle \frac{\partial \overline{u_1}~ \overline{u_3}}{\partial x_3}\right\rangle - \left\langle \frac{\partial ( \overline{u'_1 u'_3}+\overline{\widehat{u''_1u''_3}})}{\partial x_3} \right\rangle =0. \end{aligned}$$

Following Yuan and Piomelli (2014), and considering Eq. 5 and using the CSA to substitute the ISA process, Eq. 6 can be written as,

7 $$\begin{aligned}&-\,\frac{\partial \left\langle P \right\rangle _c}{\rho \partial x_1} -\left\langle \frac{\partial \tilde{p}}{\rho \partial x_1}\right\rangle _c +\nu \frac{\partial ^2 \left\langle \overline{u_1}\right\rangle _c}{\partial x_3^2} \nonumber \\&\quad +\,\nu \left\langle \frac{\partial ^2 \tilde{u}_1}{\partial x_i^2}\right\rangle _c -\frac{\partial \left\langle \tilde{u}_1\tilde{u}_3\right\rangle _c}{\partial x_3} -\frac{\partial \left\langle \overline{u'_1 u'_3}+\overline{\widehat{u''_1u''_3}} \right\rangle _c}{\partial x_3} = 0, \end{aligned}$$

where the first term is the constant pressure gradient, the second and fourth terms are respectively the form (pressure) and viscous drag forces imposed by the solid parts (e.g. Wilson and Shaw 2007; Raupach and Shaw 1982; Yuan and Piomelli 2014), the third term is the viscous shear due to the vertical gradient of the time- and spatially- averaged velocity, the fifth term is the dispersive shear stress, and the last term is the turbulence shear stress.

**Fig. 2:**
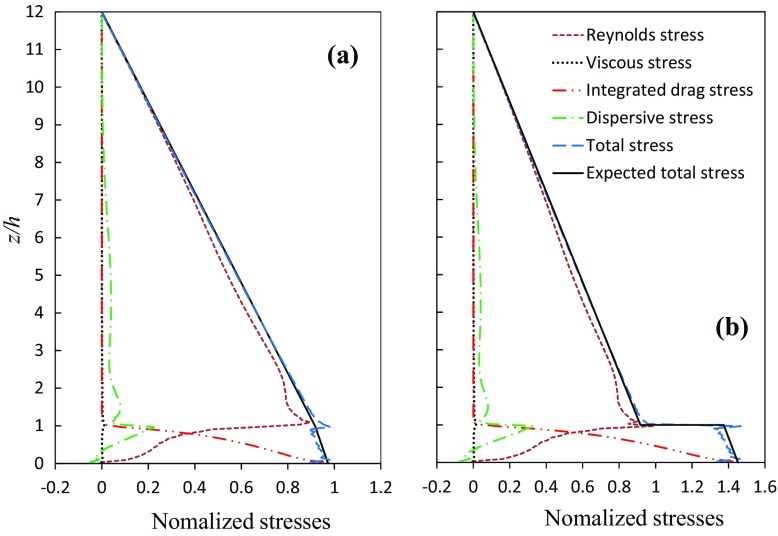
Vertical profiles of time- and spatially-averaged total shear stress, Reynolds shear stress, dispersive stress, integrated drag stress that were converted from integrated drag calculated from the top of the cuboid height, viscous shear stress, and expected total stress that was computed from the imposed driving body force from the top of the domain. All stresses are normalized by $-L_z \delta _{i1}\frac{\partial }{\partial x_i}\left( \frac{P}{\rho }\right)$, where $\delta _{i1}\frac{\partial }{\partial x_i}\left( \frac{P}{\rho }\right)$ is the specified constant pressure gradient in the fluid region. *h* is the block height. **a** CSA; **b** ISA

It is not trivial to calculate the second and fourth terms in Eq. 7, and the case shown in Fig. 1, we discretized the block height evenly into 16 horizontal slices with a thickness *h* / 16. The pressure and viscous drag forces on the cuboid surfaces within each slice were converted into an increment of shear stress over that slice. The integration of these increments from the cuboid top to a certain height is the integrated drag contribution (i.e. ‘integrated drag stress’ in Fig. 2) to the effective total time- and spatially-averaged shear stress at that height, denoted by ‘total shear stress’ or ‘total stress’.

We plot vertical profiles of the stresses rather than vertical gradients as the terms in Eq. 7, because the former shows a more evident shape, such as the total shear stress in a linear profile above the canopy given that a constant driving body force in imposed. A body force is often used to take into account the effects of buildings applied in various layers in the one-dimensional column models (e.g. Martilli 2002; Kondo et al. 2005; Husain et al. 2013). These body forces can be calculated from an estimated vertical profile of the integrated drag stress.

Figure 2 shows vertical profiles of the time- and spatially-averaged total shear stress, Reynolds shear stress, dispersive stress, integrated drag stress, viscous stress, and expected total stress computed from the imposed driving body force from the top of the domain. The expected total shear stress at height *z* is calculated as,

8a $$\begin{aligned}&(L_z-\hbox {max}(z,h)+\hbox {max}(0,h-z)(1-\lambda _p))\left( -\frac{\partial P}{\rho \partial x}\right) , \end{aligned}$$

for CSA,

8b $$\begin{aligned}&(L_z-\hbox {max}(z,h)+\hbox {max}(0,h-z)(1-\lambda _p))\left( -\frac{\partial P}{\rho \partial x}\right) \Big / \nonumber \\&\quad (1-\hbox {max}(0,\hbox {sign}(h-z))\lambda _p), \end{aligned}$$

for ISA, where $L_x$, $L_y$ and $L_z$ are the dimensions of the computational domain, *z* is the vertical coordinate, *h* is the height of the cubes, $\lambda _p$ is the packing density, max() is the max function that returns the largest value from the numbers provided, and sign() is the sign function that returns the sign of the number provided. Equation 8b confirms that the expected total shear stress for the ISA method has an abrupt increase at the cube height *h* (see Fig. 2), whereas Eq. 8a confirms that it has no abrupt change at $z=h$ for the CSA method.

Figure 2a shows data from the CSA approach. The vertical profile of the expected total stress below the cuboid height shows a slightly slower change of the slope than above the canopy as a constant body force is applied in the fluid region only and the ratio of the fluid volume to the total volume within the canopy is 67%. The viscous stress is negligible except near the cuboid height and near the floor surface, while the dispersive stress immediately below the cuboid height is much greater than that above the canopy. The contribution of the integrated drag stress to the total shear stress is dominant below $z/h = 0.5$. The time and spatial averages of the CSA data are about 30% less than for the ISA data (see Fig. 2b) because the packing density is 33%. The total stress in Fig. 2a matches very well with the expected total stress, except for a small peak at the cuboid height owing to the finite number of the discretised slices over the cuboid height. The normalized maximum total stress is slightly less than unity again because the constant pressure gradient $-\delta _{i1}\frac{\partial }{\partial x_i}\left( \frac{P}{\rho }\right)$ was applied in the fluid region only. For a greater domain height to block-height ratio, the normalized maximum total shear stress would be closer to unity.

Figure 2b shows vertical profiles of normalized shear stresses that were obtained in the same way as those in Fig. 2a except that the ISA approach is used throughout. The total stress profile shows a significant abrupt change at the cuboid height, where there is an evident discontinuous point. This is simply because the average area, which is used to calculate the stress with unit $\hbox {m}^2$ $\hbox {s}^{-2}$, changes by 33% at the cuboid height while the total shear force with unit $\hbox {m}^4$ $\hbox {s}^{-2}$ varies continuously across the entire height of the domain. In other words, the evident discontinuity in the ISA profiles is because of the discontinuity in the geometry of the solid part at the canopy height. In contrast, using the CSA approach, this issue does not occur. Figure 2a shows a continuous change of the total shear stress where the average area is constant over the entire domain height $L_z$. In Fig. 2b the profile of the expected total stress shows a significant abrupt change at the cuboid height as well; again Eq. 8 confirms this.

It is to be noted that the most crucial implication is that the ISA yields overestimated shear stresses and momentum sinks (drag) in a one-dimensional column urban model, if such a model does not take account of the solid parts of the canopy. A plot of a vertical profile of the body force implemented in the one-dimensional column model is unlikely to show this potential issue. For realistic urban geometry (e.g. Xie and Castro 2009) where the ratio of solid area to fluid area at a horizontal plane decreases gradually to zero at the canopy top, such a ‘discontinuous’ or ‘step change’ point of the vertical profiles of stresses does not occur.

Figure 2b shows that the normalized maximum shear stress at the floor is approximately 1.5, which is far greater than unity than would be expected for the ISA approach. Again, this is because the total shear force over the entire plane at various height changes continuously while the average area changes by 33% at the cuboid height. For canopies with larger packing densities, larger differences in the total shear stress within the canopy between the ISA and CSA estimates would be expected. For example, the DAPPLE site in central London (e.g. Xie and Castro 2009) has a packing density of about 50%, which is typical of city centres. More attention needs to be paid to the dispersive stress, since it is calculated from a spatial average, where an inappropriate spatial average may lead to a seriously biased interpretation of the dispersive stress. However, because it is difficult to obtain a drag distribution over the building height and the momentum flux budget, potential errors due to the ISA approach for a complex urban geometry are more difficult to identify than for a simple idealized geometry.

In summary, the main potential issue in using the ISA approach for parametrization of a one-dimensional column urban model is the overestimated magnitude of the normalized total shear stress, Reynolds shear stress, dispersive shear stress, drag coefficient, heat sources and scalar sources within the canopy.

It is interesting that these problems have not received much attention previously. The first reason might be that the spatially-averaged stresses were mainly used to parametrize flows within and above the one-dimensional plant canopy, for which the packing density is much smaller than for urban geometry, and with a ratio of the solid part to the fluid part at a given horizontal plane changing gradually to zero at the canopy top. The second reason might be that for urban flows over idealized geometries, smaller packing densities is usually used, such as 25% (e.g. Coceal et al. 2006; Xie et al. 2008). This leads to smaller differences in values calculated by the ISA and CSA approaches. The third reason might be that only recently has the parametrization of urban flows for one-dimensional column models with a few grid points within the canopy become of particular interest.

## Conclusions and Discussion

Parametrization of urban flows within the canopy is of great interest. In particular, more recently the use of one-dimensional multilayer models in mesoscale models are being developed. The Note addresses the risk of using intrinsic spatial averaging (ISA) in the processing of building-resolved computation data to supply parametrization of the canopy flows in such models.

Using a simple test case with a packing density of 33%, we have demonstrated that ISA yields greater total shear stress within the urban canopy and a more abrupt change of total shear stress at the canopy height, compared to comprehensive spatial averaging (CSA). We emphasize that this is an issue only if the urban column model does not account explicitly for the volume of the buildings, and we trust that no present multilayer urban column model contains such an error.

The CSA results confirm that the total vertical momentum flux of the entire domain (i.e. the total shear force) is continuous within and above the urban canopy, regardless of the packing density, or whether the urban geometry changes abruptly. The CSA approach is useful for extremely complex scenarios, such as in a plant canopy or in porous material, where it might be difficult to estimate the solid volume, and it would be more sensible to calculate the shear stresses based on the total area including both fluid and solid parts. The CSA approach is also useful in calculating the global shear stresses over irregular rough walls where details of the roughness elements are not able to be resolved. Usually the total area of a plane including solid and the fluids part is used to calculate the shear stresses.

One-dimensional column models of urban flows will benefit from the CSA approach, where an accurate estimation of the body forces (i.e. the drag which takes into account the effects of buildings) imposed at various layers in these models is crucial. It is to be noted that the ‘typical’ means of estimating the body force is to use the mean wind speed within the canopy and a drag coefficient, which are usually difficult to obtain. The CSA approach is able to provide an alternative way. At a certain height, the body forces can be converted from the increment at that height of the vertical profile of integrated drag stress of the CSA data (Fig. 2a) given that the integrated drag stress at the ground surface is known and a vertical profile of the integrated drag stress is approximated. It is to be noted that at the ground the integrated drag stress is equal to the total shear stress (Fig. 2a), and the key is to have an appropriate estimation of the total stress at the ground. Further discussion of an approximation of the total surface shear stress and the shape of the vertical profile of the integrated drag stress is beyond the scope of this Note.

We do not imply that the ISA procedure is inappropriate; certainly the ISA has a clear physical meaning, e.g., its focus on the local fluid field within the urban canopy. If the averaging of ISA is properly done, and the parametrizations are coherent with the averaging technique chosen, then the global momentum conservation for a one-dimensional column model is satisfied. On the other hand, the CSA implicitly satisfies the global momentum conservation. Although we would not recommend replacing the ISA with the CSA entirely, we suggest that the CSA is preferred if a parametrization is needed for one-dimensional column urban models.

We have focused on time- and spatially-averaged shear stresses (i.e. vertical momentum fluxes) in urban environments for one-dimensional column urban models, and for dealing with heat and passive scalar fluxes, the CSA approach should have the same benefit as dealing with the momentum fluxes. It is most important to consider the time- and spatially-averaged vertical fluxes where one-dimensional urban models are concerned. In some applications volume-average quantities obtained from the ISA approach are also of interest. Nevertheless, if an estimation of the local velocities, temperature and scalar concentration, within the urban canopy is of greater interest, only building-geometry resolved models (such as computational fluid dynamics models) are able to give a reasonable estimation of these quantities. This can be achieved by coupling with mesoscale models, but at a very high computational cost.
